# Appearance-related social media behavior and eating disorder pathology: evidence from the german social media appearance preoccupation scale in nonclinical and clinical samples

**DOI:** 10.1186/s40337-026-01762-z

**Published:** 2026-09-05

**Authors:** Sebastian Brand, Isabelle Kappelt, Kai Kloberdanz, Melanie J. Zimmer-Gembeck, Stefanie M. Jungmann

**Affiliations:** 1 Department of Clinical Psychology and Psychotherapy of Childhood and Adolescence, Johannes Gutenberg-University Mainz, Mainz, Germany; 2 School of Applied Psychology, Griffith University, Queensland, Australia; 3 Griffith Centre for Mental Health, Griffith University, Queensland, Australia

**Keywords:** Social media, Body image, Eating disorders, Scale validation

## Abstract

**Background:**

Image- and video-based social media have been linked to body dissatisfaction and eating pathology, partly through increased appearance-based social comparison and self-focused attention. The Social Media Appearance Preoccupation Scale (SMAPS) assesses three behaviors relevant in this context: Online Self-Presentation, Appearance-Related Activity, and Appearance Comparison. Higher scores on these dimensions have been linked to more intensive social media use, increased anxiety, depressive symptoms, and disordered eating. However, research has largely relied on English-speaking, non-clinical samples. The present studies therefore aimed to translate and validate a German version of the SMAPS and to compare appearance-related social media behaviors between individuals with eating disorders and healthy controls.

**Methods:**

The scale was translated in a systematic translation-back-translation procedure and then examined in two cross-sectional studies. In Study 1, *N* = 363 young adults completed a survey with the aim of testing the factor structure of the German SMAPS and its associations with eating disorder pathology, general psychological distress, and problematic internet use. In Study 2, multivariate analysis of variance was used to compare German SMAPS scores between *n* = 21 outpatients with diagnosed eating disorders and *n* = 37 healthy controls.

**Results:**

The three-factor structure of the German SMAPS showed acceptable model fit. All subscales exhibited good internal consistency (ω = 0.83-0.88). Of the three dimensions, Appearance Comparison was most strongly associated with eating disorder pathology and showed the most consistent associations with psychological distress and problematic internet use. It also significantly differentiated patients with eating disorders from healthy controls (*p* = .004, η²_p_ = 0.14, 90% CI [0.03, 0.29]).

**Conclusions:**

The German SMAPS is a reliable and valid tool for assessing appearance-related social media behaviors in German-speaking populations. The findings suggest that the three dimensions may differ in clinical relevance, with appearance comparison warranting particular attention in future research.

**Supplementary Information:**

The online version contains supplementary material available at https://doi.org/10.1186/s40337-026-01762-z.

## Abstract (plain language summary)

Social media platforms encourage users to focus on how they and others look. This can include editing photos, comparing one’s appearance to others, and monitoring reactions to posts. These behaviors may negatively affect how people feel about their bodies and how they eat. We translated a questionnaire called the Social Media Appearance Preoccupation Scale (SMAPS) into German and tested it in two studies. In the first study, 363 young adults completed the questionnaire along with measures of disordered eating, psychological symptoms, and internet use. In the second study, we compared 21 patients with eating disorders to 37 healthy individuals. The questionnaire worked as intended in German and captured three types of behavior: presenting oneself online, engaging with appearance-related content, and comparing one’s appearance to others. Among these, appearance comparison was most strongly linked to eating problems and psychological distress. Patients with eating disorders scored higher on appearance comparison than healthy individuals. These findings suggest that it is not just how much people use social media that matters, but specifically how they engage with appearance-related content.

**Supplementary Information:**

The online version contains supplementary material available at https://doi.org/10.1186/s40337-026-01762-z.

## Background

The use of social media platforms has become an integral part of everyday life for a large proportion of the global population. Currently, more than half of the world’s population uses social media, and global adoption continues to increase steadily [1]. This development has been accompanied by growing concern regarding potential negative psychological consequences, leading to substantial research interest in internet use in general and social media use as part of it [2]. Meta-analytic findings based primarily on global indicators of social media use (e.g., time, frequency) show that more use has small associations with poorer mental health (e.g., depressive symptoms) [3]. However, the robustness and practical significance of these small associations have been questioned due to substantial heterogeneity and methodological limitations across studies [4]. Nevertheless, among the various mental health domains discussed in this context, findings regarding the association between social media use and body dissatisfaction appear more consistent. This is supported by meta-analyses from cross-sectional [5] as well as longitudinal and experimental studies [6]. Although traditional mass media have long been recognized as influential in shaping body image, social media may exert a particularly strong impact due to their interactive, visual, and personalized nature [7, 8].

Social media platforms differ widely in content format (e.g., text, images, videos) and user functions (e.g., posting, commenting, rating), yet they share a defining feature: users are not merely passive consumers but active producers and evaluators of content [9]. Unlike traditional media, social media allow individuals to curate their own appearance through photo selection, editing tools, and filters, to receive immediate feedback in the form of likes and comments, and to engage in direct social comparison with peers and influencers [10–13]. Additionally, algorithm-driven content curation may further amplify exposure to appearance-focused material by selectively presenting content aligned with users’ prior behavior and preferences [14, 15].

The positive association between media exposure and body dissatisfaction is consistent with the Tripartite Influence Model [16]. According to this model, sociocultural influences (e.g., peers, parents, media) promote the internalization of appearance ideals and encourage appearance-based social comparisons. Drawing on Social Comparison Theory [17], such comparisons are particularly detrimental when the ideals are unrealistic and unattainable, frequently resulting in body dissatisfaction [18]. This model has been supported by research in the context of traditional media [19, 20], and evidence suggests that key mechanisms of the model (i.e., internalization and appearance-based social comparison) also operate within social media environments [21–25].

Importantly, research indicates that it is not the social media use per se that is most relevant for body image outcomes, but rather how social media is used and which content individuals engage with [26, 27]. For example, image-based platforms such as Instagram elicit more appearance-based social comparisons and higher body dissatisfaction than primarily text-based platforms [28]. Furthermore, within image-based platforms, appearance-related activities, such as viewing idealized images, engaging with fitness or weight-loss content (i.e., *fitspiration* or *thinspiration*), and posting or editing selfies are particularly strongly associated with body dissatisfaction [26, 27, 29–32]. In addition to social comparison processes, self-objectification has been identified as a mechanism linking media exposure to body image disturbances. *Objectification Theory* posits that repeated exposure to the sexualized and objectified portrayal of bodies leads individuals to internalize an observer’s perspective on their own bodies, resulting in chronic body surveillance [33]. On social media, this process is especially pronounced due to the emphasis on visual self-presentation and continuous evaluative feedback. Activities such as monitoring likes and anticipating others’ evaluations reflect heightened body surveillance and have been linked to body dissatisfaction, body shame, and disordered eating [34–36].

Given the differentiated nature of social media use, the assessment of mere usage time has been increasingly criticized as insufficient [37]. To address this gap, Zimmer-Gembeck and colleagues [38] developed the Social Media Appearance Preoccupation Scale (SMAPS), a measure designed to assess appearance-related social media behaviors. Based on factor-analytic findings and prior literature, the SMAPS captures three core dimensions: Online self-presentation, Appearance-Related Activity, and Appearance Comparison. Importantly, the SMAPS does not merely assess the extent of appearance-related social media use but also captures cognitive and emotional aspects of appearance preoccupation. Initial findings showed that higher SMAPS scores are associated with greater body image concerns, elevated anxiety and depressive symptoms, greater integration of social media into daily life, and disordered eating, with young women reporting higher scores than young men [38]. Subsequent studies have replicated and extended these findings across cross-sectional and longitudinal designs [39–42]. However, existing evidence on the SMAPS is largely based on English-speaking, non-clinical samples. To the authors’ knowledge, no German version of the SMAPS is available, and findings based on clinical populations, particularly individuals with diagnosed eating disorders, are currently lacking.

### The present studies

The present manuscript comprises two consecutive studies. The aim of the first study was to test the psychometric properties of the German SMAPS by examining its factor structure, internal consistency, and associations with eating disorder symptomatology, psychopathological distress, and problematic internet use. To this end, it was hypothesized that a confirmatory factor analysis (CFA) on the original three-factor structure of the SMAPS would show at least acceptable model fit and the factors would have good internal consistency. Consistent with previous research, higher SMAPS scores in women than men were expected, while correlations with age were explored in the analysis as well. Furthermore, it was expected that higher SMAPS scores would be associated with higher eating disorder symptoms, higher levels of psychopathological distress, and more problematic internet use. The second study aimed to investigate whether SMAPS scores differ between individuals with diagnosed eating disorders and healthy control participants. It was hypothesized that individuals with eating disorders would report higher levels of Online Self-Presentation, Appearance-Related Activity, and Appearance Comparison, compared to healthy controls. Furthermore, Study 1 associations between SMAPS scores and eating disorder symptomatology and psychopathological distress were to be cross-validated in Study 2.

## Study 1

### Method

#### Participants

Overall, *N* = 492 individuals accessed the questionnaire. After exclusion of those that did not meet the inclusion criteria (see below), provided incomplete data, or showed implausible response patterns (e.g., no variation across items), the final sample consisted of *N* = 363. Participants ranged from 18 to 64 years of age with a mean of *M* = 26.6 (*SD* = 7.9). Further sociodemographic information is depicted in Table 1. Most frequently reported reasons for posting content online were: informing others (59.0%), positive self-representation (44.6%), and maintaining a social media presence (39.1%). About one quarter reported posting to improve self-esteem (25.6%), to feel better (22.6%), or to influence others’ impressions about oneself (28.4%).

**Table 1:** Sociodemographic Characteristics for the samples of Study 1 (*N* = 363) and Study 2 (*N* = 58)

Participant Information	*Study 1*		*Study 2*			
			*ED*		*HC*	
	*n*	*%*	*n*	*%*	*n*	%
*Gender*						
Men	50	13.8	1	4.8	4	10.8
Women	309	85.1	20	95.2	33	89.2
Non-Binary	4	1.1	-	-	-	-
*Age*						
18–24	177	48.8	8	38.1	4	10.8
25–34	152	41.9	6	28.6	29	78.4
35+	34	9.4	7	33.3	4	10.8
*Education*						
No degree	1	0.3	-	-	-	-
Lower school	7	1.9	-	-	-	-
Intermediate school	9	2.5	4	19.0	5	13.5
Higher Education Entrance	154	42.4	7	33.3	6	16.2
Bachelor’s degree	148	40.8	3	14.3	16	43.2
Master’s degree	40	11.0	5	23.8	10	27.0
Doctorate	3	0.8	-	-	-	-
Other^a^	1	0.3	2	9.5	-	-
*Occupation*						
School student	1	0.3	1	4.8	-	-
Vocational training	3	0.8	-	-	4	10.8
University student	255	70.2	6	28.6	19	51.4
Employee/Self-Employed	90	24.8	12	57.1	14	37.8
Unemployed / Retired	10	2.9	1	4.8	-	-
Parental Leave	2	0.6	1	4.8	-	-
Other^b^	2	0.6	-	-	-	-
*Channels*						
Instagram	328	90.4	19	90.5	36	97.3
Youtube	164	45.2	15	71.4	25	67.6
Snapchat	160	44.1	8	38.1	16	43.2
Facebook	152	41.9	7	33.3	11	29.7
Whatsapp Status	126	34.7	9	42.9	16	43.2
TikTok	67	18.5	8	38.1	11	29.7
X/Twitter	38	10.5	2	9.5	2	5.4
Other^c^	30	8.3	1	4.8	3	8.1
*Posting Frequency*						
Once per month	205	56.5	4	19.0	11	29.7
Less than once per week	90	24.8	6	28.6	10	27.0
Two to three times per week	48	13.2	2	9.5	2	5.4
Four to five times per week	9	2.5	1	4.8	1	2.7
Daily	11	3.0	-	-	-	-
No posts in the last three months	-	-	8	38.1	13	35.1

ED = Eating Disorder; HC = Healthy Control; ^a^Other included master craftsman qualifications; ^b^Other included au pair and voluntary social service; ^c^Other included BeReal, LinkedIn/Xing, Pinterest, Reddit, Twitch, Tumblr and Telegram/Signal/iMessage

#### Procedure

Data was collected in two independent waves (June-September 2020; May-August 2023), using the online survey platform SoSciSurvey [43]. We aimed to recruit *N* = 500 participants, a sample size that is typically regarded as sufficient for CFA [44]. Subsequent Monte-Carlo analysis [45] based on the factor structure reported by Zimmer-Gembeck and colleagues [38] using 1,000 replications and weighted least squares mean and variance adjusted estimation (WLSMV), further confirmed that this sample size would provide sufficient statistical power. Participants gave active, informed consent prior to completing a survey that took approximately 15–20 min and did not involve any follow-up assessment. Participants were invited to enter a raffle for three vouchers for an online bookstore with a value of €20 each for compensation. Psychology students had the option to receive course credit. Participants were recruited via social media platforms and university mailing lists. Inclusion criteria were an age of at least 18 years, sufficient German language proficiency, and active social media use, defined as having posted content at least once per month during the preceding three months.

#### Measures

A systematic translation-back-translation procedure [46] was conducted with the original SMAPS [38]. The initial translation was completed by a native German speaking psychologist and subsequently back-translated by a bilingual translator with native English proficiency. All original items were retained (see Supplementary Material 2). The 18 items capture three factors: *Online Self-Presentation* (OS; 7 items), *Appearance-Related Activity* (AA; 5 items), and *Appearance Comparison* (AC; 6 items). Items such as *When others upload photos of me to social media*,* I focus on whether I looked good* are rated on a 7-point Likert scale (1 = *strongly disagree* to 7 = *strongly agree*), consistent with the original version.

The Eating Disorder Examination-Questionnaire 8 (EDE-Q8) [47], an eight-item short form of the German Eating Disorder Examination-Questionnaire [48], assesses eating disorder pathology by self-report. The eight items assess *Restraint*,* Eating Concern*,* Shape Concern*, and *Weight Concern* during the past 28 days, rated on a 7-point Likert scale ranging from 0 to 6 with item-specific anchors (e.g., 0 = *no days* to 6 = *every day*). Internal consistency was excellent (ω = 0.93).

General psychological distress was assessed using the Brief Symptom Inventory-18 (BSI-18) [49, 50]. The BSI-18 uses three subscales: somatization, anxiety, and depression, each consisting of six items. All three subscales exhibited acceptable to good internal consistency (ω_SOM_ = 0.79, ω_DEP_ = 0.88, ω_ANX_ = 0.85). Responses are rated on a 5-point Likert-scale from 0 (*not at all*) to 4 (*very much*). Scores can be summarized as a Global Severity Index (GSI), which showed excellent internal consistency (ω = 0.93).

The Generalized Problematic Internet Use Scale 2 (GPIUS-2) [51] in its German version [52] assesses aspects of problematic internet use. The scale consists of five subscales: *Preference for Online Social Interaction* (POSI), *Mood Regulation* (MR), *Cognitive Preoccupation* (CP), *Compulsive Use* (CU), and *Negative Outcome* (NO), which showed acceptable to good internal consistencies (ω_POSI_ = 0.85, ω_MR_ = 0.77, ω_CP_ = 0.74, ω_CU_ = 0.85, ω_NO_ = 0.69). Responses are given on a 5-point Likert scale (1 = *strongly disagree* to 5 = *strongly agree*). Internal consistency of the total score was good (ω = 0.86).

Additionally, four single-item questions assessed participants’ engagement with their own posts: (a) overall frequency of engaging with own posts, (b) intensity of engagement in editing images or videos, (c) intensity of engagement in selecting images or videos, and (d) intensity of monitoring others’ reactions to own posts. All items were rated on a 5-point Likert scale ranging from 1 (very rarely/not at all) to 5 (very frequently/very strongly).

#### Data analysis

Statistical analyses were conducted using R version 4.5.1 [53] with the packages *psych*, *dplyr*, *lavaan*, and *effectsize*. First, the factor structure of the SMAPS was examined using a CFA. The three-factor structure proposed by Zimmer-Gembeck and colleagues [38] was specified, with each item constrained to load on only one factor and all residual covariances fixed to zero. WLSMV estimation was applied to account for the ordinal nature of Likert scales [54]. Following commonly applied cutoff criteria, model fit was evaluated using the Root Mean Square Error of Approximation (RMSEA; ≤ 0.05 good fit, ≤ 0.08 acceptable fit), the Standardized Root Mean Square Residual (SRMR; ≤ 0.05 good fit, ≤ 0.10 acceptable fit), as well as the Comparative Fit Index and the Tucker-Lewis Index (CFI and TLI; ≥ 0.90 acceptable fit, ≥ 0.95 good fit) [55–57]. Internal consistency of the SMAPS and its subscales was assessed using McDonald’s omega (ω), interpreted along conventional guidelines for internal consistency coefficients (≥ 0.70 acceptable, ≥ 0.80 good, ≥ 0.90 excellent) [58]. Male and female SMAPS scores were compared using Welch’s *t*-test, which does not assume equal variances and is therefore appropriate for unequal group sizes. Thus, the resulting degrees of freedom vary across comparisons. Non-binary participants were not included in these comparisons due to the small number of participants in this group (*n* = 4). Associations between the SMAPS subscales, age, and scores on the EDE-Q8, BSI-18, GPIUS-2, as well as the four items measuring engagement with online posting, were examined using two-tailed Pearson correlation analyses. Bonferroni correction was applied to account for multiple testing [59]. For correlation coefficients and effect sizes, two-sided confidence intervals are reported.

### Results

#### Confirmatory factor analysis

**Fig. 1:**
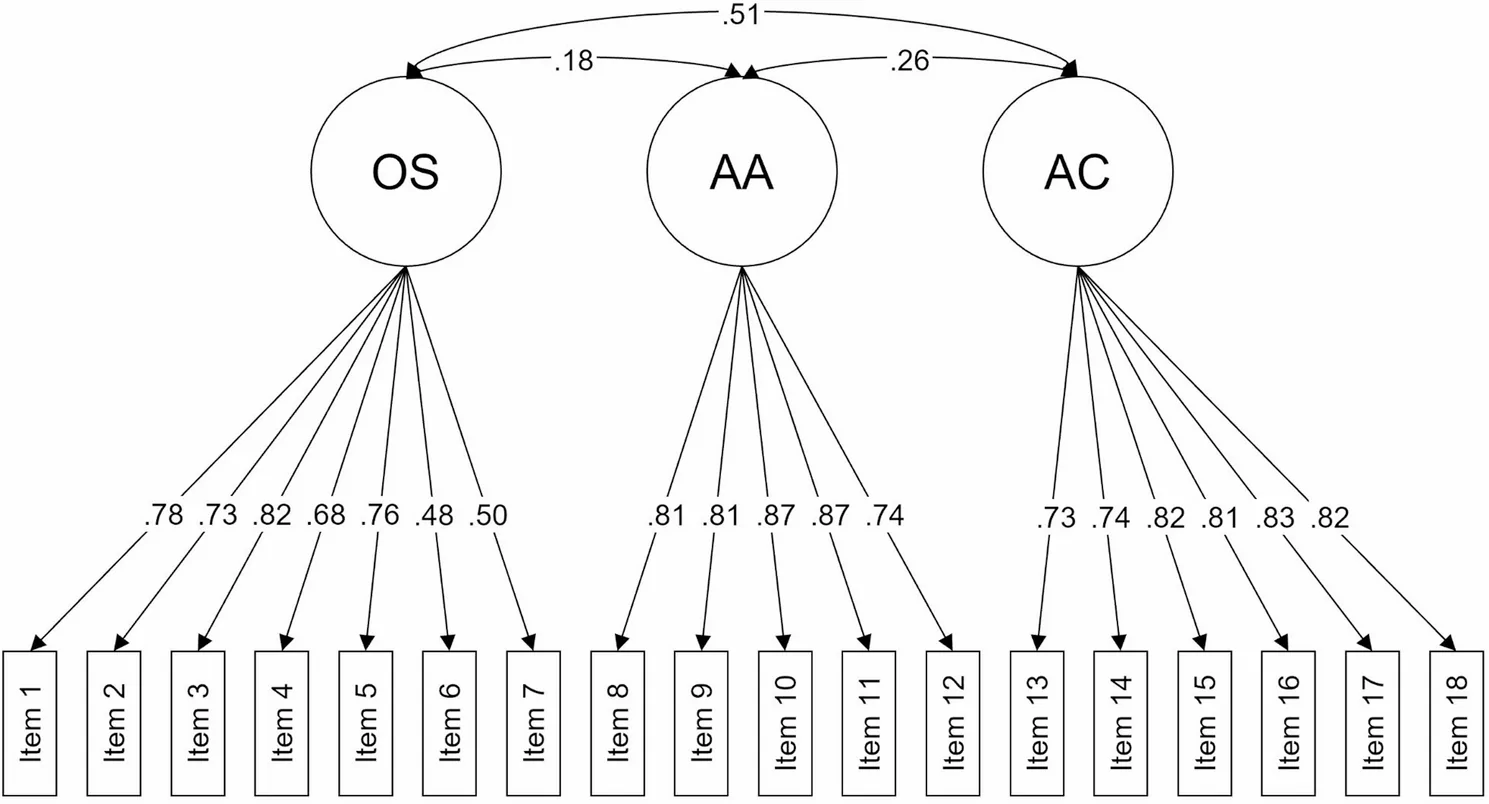
Three Factor Model of the German Social Media Appearance Preoccupation Scale. OS = Online Self-Presentation; AA = Appearance-Related Activity; AC = Appearance Comparison; Circles represent latent variables, squares refer to manifest variables, single-headed arrows represent standardized factor loadings, and double-headed arrows between latent variables represent standardized latent correlations; error terms of manifest variables are not shown; all factor loadings and latent correlations are significant at *p* < .001

The three-factor model (see Fig. 1) showed acceptable fit on most indices, χ²(132) = 515.04, *p* < .001, RMSEA = 0.090, 90% CI [0.081, 0.098], CFI = 0.943, TLI = 0.934, SRMR = 0.071. All standardized factor loadings were significant and ranged from λ = 0.48-0.82 for Online Self-Presentation, λ = 0.74-0.87 for Appearance-Related Activity, and λ = 0.73-0.83 for Appearance Comparison. The three factors were significantly intercorrelated, *r* = 0.18 to 0.51, with the highest correlation between Online Self-Presentation and Appearance Comparison. Following the original validation [38], an additional model was estimated exploratorily, freeing the four residual covariances with the highest modification indices. This model showed improved fit, χ²(128) = 263.15, RMSEA = 0.054, 90% CI [0.045, 0.063], CFI = 0.980, TLI = 0.976, SRMR = 0.055 (see Supplementary Material 1, Supplementary Figure S1). The three SMAPS scales showed good internal consistency, ω_OS_ = 0.83, ω_AA_ = 0.87, and ω_AC_ = 0.88.

#### Associations with demographic variables and related constructs

Descriptively, subscale means of the SMAPS were *M* = 4.5 (*SD* = 1.2) for Online Self-Presentation, with a skewness of -0.66 and a kurtosis of 0.51, *M* = 2.3 (*SD* = 1.3) for Appearance-Related Activity, with a skewness of 0.95 and a kurtosis of -0.02, as well as *M* = 3.6 (*SD* = 1.5) for Appearance Comparison, with a skewness of 0.20 and a kurtosis of -0.90.

The subscales Online Self-Presentation, *r*(361) = − 0.27, *p* < .001, 95% CI [-0.36, − 0.17], and Appearance Comparison, *r*(361) = − 0.24, *p* < .001, 95% CI [-0.34, − 0.14], were significantly negatively related with age, indicating younger participants showing higher scores on the respective scales. For Appearance-Related Activity this trend was non-significant, *r*(361) = − 0.09, *p* = .087, 95% CI [-0.19, 0.01]. Furthermore, women (*M* = 4.6, *SD* = 1.1) showed significantly higher scores in Online Self-Presentation, *t*(58) = -2.24, *p* = .029, *d* = -0.42, 95% CI [-0.72, -0.12], compared to men (*M* = 4.1, *SD* = 1.5). The same pattern was observed for Appearance Comparison, with women (*M* = 3.7, *SD* = 1.5) showing higher scores than men (*M* = 2.8, *SD* = 1.3), *t*(73) = -4.20, *p* < .001, *d* = -0.57, 95% CI [-0.87, -0.26]. In contrast, for Appearance-Related Activity men (*M* = 2.8, *SD* = 1.4) showed higher scores than women (*M* = 2.3, *SD* = 1.3), *t*(64) = 2.47, *p* = .016, *d* = 0.39, 95% CI [0.09, 0.69].

Table 2 presents the correlations between the SMAPS subscales and the EDE-Q8, BSI-18, GPIUS-2, as well as the four engagement items. A Bonferroni correction was applied to account for multiple testing (*k* = 45; 3 SMAPS scales × 15 criterion variables).

**Table 2:** *Relationship between SMAPS with EDE-Q8*,* BSI-18*,* GPIUS-2*,* and Engagement with Online Posting Items* (*N* = 363)

Variable	*OS*			*AA*			*AC*		
	*r*_(361)_	*p*	95% CI	*r*_(361)_	*p*	95% CI	*r*_(361)_	*p*	95% CI
EDE-Q8	0.27	< 0.001	[0.17, 0.36]	0.19	0.015	[0.09, 0.29]	0.69	< 0.001	[0.64, 0.74]
BSI-18 (GSI)	0.16	0.075	[0.06, 0.26]	0.16	0.123	[0.05, 0.26]	0.41	< 0.001	[0.32, 0.49]
Somatization	0.07	1	[-0.03, 0.17]	0.21	0.003	[0.11, 0.30]	0.28	< 0.001	[0.18, 0.37]
Depression	0.19	0.017	[0.08, 0.28]	0.11	1	[0.01, 0.21]	0.42	< 0.001	[0.34, 0.50]
Anxiety	0.16	0.092	[0.06, 0.26]	0.11	1	[0.01, 0.21]	0.36	< 0.001	[0.27, 0.45]
GPIUS-2	0.36	< 0.001	[0.26, 0.44]	0.18	0.034	[0.07, 0.27]	0.32	< 0.001	[0.22, 0.41]
POSI	0.10	1	[0.00, 0.20]	0.18	0.019	[0.08, 0.28]	0.23	< 0.001	[0.13, 0.33]
MR	0.31	< 0.001	[0.22, 0.40]	− 0.01	1	[-0.11, 0.09]	0.22	0.001	[0.12, 0.32]
CP	0.32	< 0.001	[0.23, 0.41]	0.19	0.011	[0.09, 0.29]	0.21	0.002	[0.11, 0.31]
CU	0.31	< 0.001	[0.21, 0.40]	0.05	1	[-0.05, 0.15]	0.23	< 0.001	[0.13, 0.33]
NO	0.20	0.007	[0.10, 0.29]	0.27	< 0.001	[0.17, 0.36]	0.24	< 0.001	[0.14, 0.33]
Engagement									
Frequency	0.29	< 0.001	[0.20, 0.39]	0.24	< 0.001	[0.14, 0.33]	0.25	< 0.001	[0.15, 0.34]
Editing	0.36	< 0.001	[0.26, 0.44]	0.23	< 0.001	[0.13, 0.33]	0.20	0.005	[0.10, 0.30]
Selection	0.38	< 0.001	[0.28, 0.46]	0.20	0.004	[0.10, 0.30]	0.25	< 0.001	[0.15, 0.35]
Monitoring	0.50	< 0.001	[0.42, 0.57]	0.12	0.866	[0.02, 0.22]	0.24	< 0.001	[0.14, 0.33]

*p* represents Bonferroni adjusted *p*-values (*p* x 45 tests), capped at 1; OS = Online Self-Presentation, AA = Appearance Related Activity, AC = Appearance Comparison; EDE-Q8 = Eating Disorder Examination - Questionnaire 8; BSI-18 = Brief Symptom Inventory-18; GSI = Global Severity Index; GPIUS-2 = Generalized Problematic Internet Use Scale 2, POSI = Preference for Online Social Interaction, MR = Mood Regulation, CP = Cognitive Preoccupation, CU = Compulsive Use, NO = Negative Outcome

##### Relationship between SMAPS and eating disorder symptomatology

All three SMAPS subscales showed significant positive correlations with eating disorder symptomatology. Online Self-Presentation, *r*(361) = 0.27, *p* < .001, 95% CI [0.17, 0.36], and Appearance-Related Activity, *r*(361) = 0.19, *p* = .015, 95% CI [0.09, 0.29], showed small correlations, whereas Appearance Comparison demonstrated a large correlation, *r*(361) = 0.69, *p* < .001, 95% CI [0.64, 0.74].

##### Relationship between the SMAPS and symptoms of psychopathology

Appearance Comparison showed a significant moderate positive correlation with general psychopathological distress, *r*(361) = 0.41, *p* < .001, 95% CI [0.32, 0.49], with the strongest association for depressive symptoms, *r*(361) = 0.42, *p* < .001, 95% CI [0.34, 0.50], followed by anxiety, *r*(361) = 0.36, *p* < .001, 95% CI [0.27, 0.45], and somatization, *r*(361) = 0.28, *p* < .001, 95% CI [0.18, 0.37]. Online Self-Presentation showed a small significant correlation with depressive symptoms, *r*(361) = 0.19, *p* = .017, 95% CI [0.08, 0.28]. For Appearance-Related Activity, only a positive correlation with somatization emerged, *r*(361) = 0.21, *p* = .003, 95% CI [0.11, 0.30].

##### Relationship between the SMAPS and problematic internet use

All three SMAPS subscales correlated significantly positively with problematic internet use, with Online Self-Presentation showing the strongest association, *r*(361) = 0.36, *p* < .001, 95% CI [0.26, 0.44], followed by Appearance Comparison, *r*(361) = 0.32, *p* < .001, 95% CI [0.22, 0.41], and Appearance-Related Activity, *r*(361) = 0.18, *p* = .034, 95% CI [0.07, 0.27]. At the subscale level, Appearance Comparison showed significant correlations with all GPIUS-2 subscales. Online Self-Presentation was associated with every subscale except Preference for Online Social Interaction. Appearance-Related Activity showed only significant associations with Negative Outcome, Cognitive Preoccupation, and Preference for Online Social Interaction.

##### Relationship between the SMAPS and engagement with online posting

All SMAPS subscales showed significant small to moderate correlations with engagement frequency (*r* = 0.24 to 0.29, all *p* < .001), intensity of editing posts (*r* = 0.20 to 0.36, all *p* ≤ .005) and intensity of selecting posts (*r* = 0.20 to 0.38, all *p* ≤ .004). For monitoring reactions to posts, only Online Self-Presentation, *r*(361) = 0.50, *p* < .001, 95% CI [0.42, 0.57], and Appearance Comparison, *r*(361) = 0.24, *p* < .001, 95% CI [0.14, 0.33], reached significance. Overall, Online Self-Presentation demonstrated descriptively the strongest associations with engagement variables.

## Study 2

### Method

#### Participants

A sample of patients with a diagnosed eating disorder (ED) and healthy controls (HC) was recruited for Study 2. Overall, 58 participants (91.4% women) completed the questionnaire. The ED group included *n* = 21 participants, while the HC group included *n* = 37 participants. No participants had to be excluded due to incomplete or implausible data, and no missing values were present across any study variable. The age range was 21–56 years (*M* = 31.6, *SD* = 11.0) for the ED group and 22–60 years (*M* = 29.3, *SD* = 7.9) for the HC group. Further demographic variables are depicted in Table 1. There was no significant difference between ED and HC regarding age, *t*(32) = -0.84, *p* = .407, *d* = -0.24, 95% CI [-0.80, 0.32], gender, χ²(1) = 0.62, *p* = .430, φ = 0.10, 95% CI [0.00, 0.36], or education, χ²(4) = 9.02, *p* = .061, *V* = 0.30, 95% CI [0.00, 0.54], as intended by a frequency-matching strategy (see below).

Within the ED group, patients were mostly diagnosed with anorexia nervosa (42.9%), followed by atypical anorexia nervosa (19.0%) and binge eating disorder (19.0%), as well as bulimia nervosa (9.5%) and unspecified eating disorder (9.5%). Among participants who indicated posting at least occasionally (*n* = 37) in the overall Study 2 sample, the most frequently reported reasons for posting were informing others (59.5%), maintaining a social media presence (56.8%), and positive self-representation (40.5%). About a third of participants reported posting to improve self-esteem (35.1%) or to feel better (29.7%), and about a fifth to influence others’ impressions about oneself (21.6%).

#### Procedure

Data for Study 2 was collected in two independent waves (May-August 2023; June-September 2025). An a priori power analysis using the *pwr* package [60], targeting a medium effect size (*f*² = 0.15) with 1-β = 0.80 and α = 0.05, recommended a sample size of *N* = 77 in total (about *n* = 39 per group). The general process was similar to Study 1.

Recruitment of the ED sample was conducted in collaboration with therapists from a specialized eating disorders unit of an outpatient psychotherapy clinic. Eligibility criteria were identical to those of Study 1, with the additional requirement of an eating disorder diagnosis. Additionally, in contrast to Study 1, social media use in Study 2 was not restricted to active posting behavior to avoid unnecessary exclusion of clinical participants and to capture appearance-related preoccupation also among passive users. The HC group was recruited via social-media posts and mailing lists with the aim of frequency-matching this sample to the ED group with respect to age, gender, and education. Inclusion criteria were the same, except for the absence of an eating disorder.

#### Measures

The measures from Study 1 were also included in Study 2, except for the GPIUS-2. Specifically, participants completed the German version of the SMAPS (ω_OS_ = 0.84, ω_AA_ = 0.88, and ω_AC_ = 0.94), the EDE-Q8 (ω = 0.94), the BSI-18 (ω_Som_ = 0.88, ω_Dep_ = 0.91, and ω_Anx_ = 0.89, ω_GSI_ = 0.95), and the four single items measuring engagement with online posting. In the overall Study 2 sample, all scales and their respective subscales demonstrated good to excellent internal consistency.

#### Data analysis

Statistical analyses for Study 2 were conducted using R version 4.5.1 [53] with the packages *psych*, *dplyr*,* car*,* emmeans* and *effectsize*. Group differences on the three SMAPS subscales were examined using a one-way Multivariate Analysis of Variance (MANOVA). As a subsequent analysis, a one-way Multivariate Analysis of Covariance (MANCOVA) with the GSI as covariate was conducted to examine whether potential group differences persist after controlling for general psychopathology. To cross-validate the findings from Study 1, two-tailed Pearson correlations between the SMAPS subscales and EDE-Q8, BSI-18, as well as personal engagement with online posts (for those who indicated active posting), were computed for the combined sample, again using Bonferroni correction to account for multiple testing. For correlation coefficients and effect sizes, two-sided confidence intervals are reported.

### Results

#### Group comparison

**Fig. 2:**
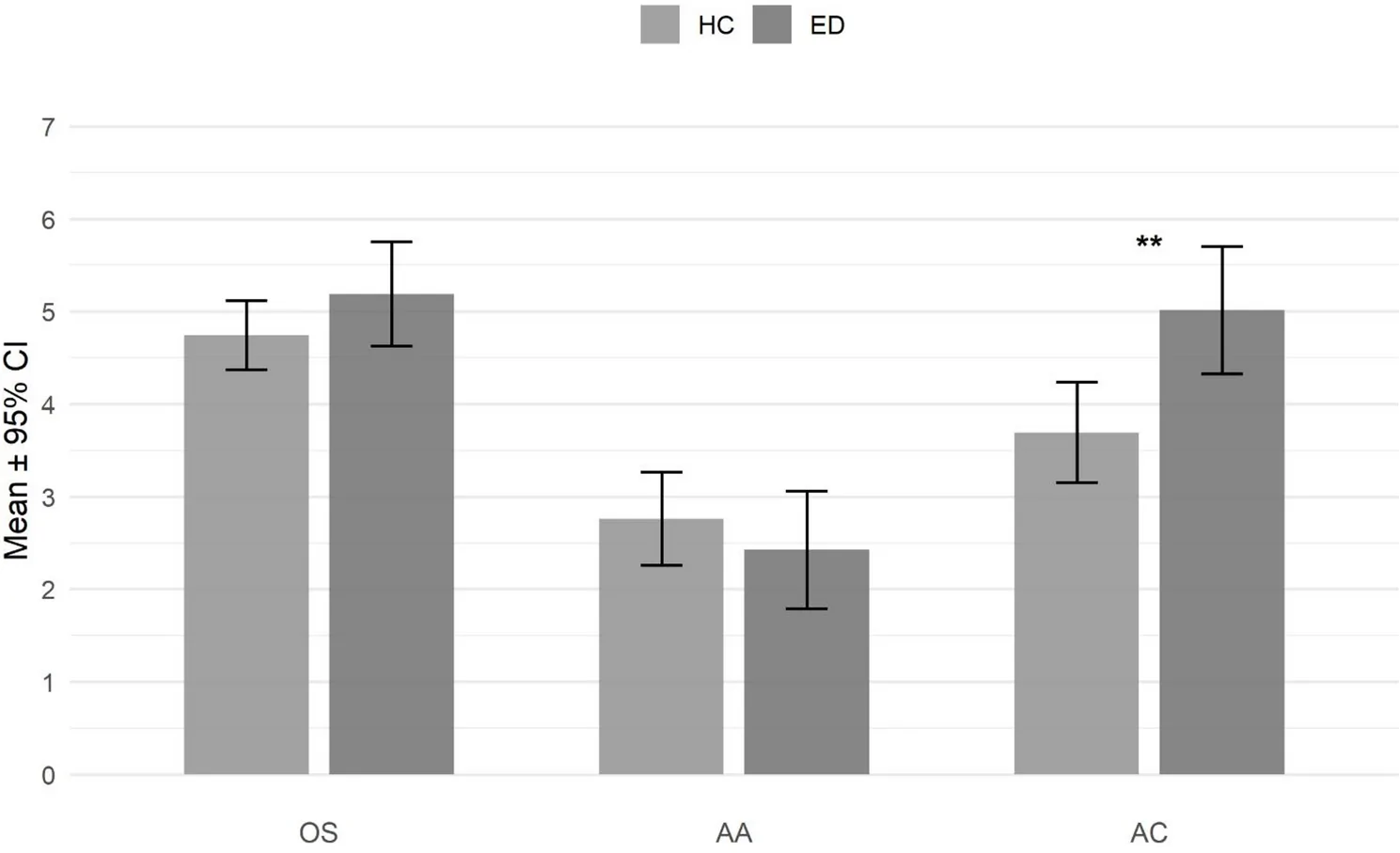
Means of the SMAPS subscales by group. OS = Online Self-Presentation; AA = Appearance-Related Activity; AC = Appearance Comparison; HC = Healthy Controls (*n* = 37); ED = Eating Disorder Group (*n* = 21). Error bars represent 95% confidence intervals; **p < .01

The one-way MANOVA revealed a significant multivariate effect of group on the SMAPS subscales, *F*(3, 54) = 4.51, *p* = .007, η²_p_ = 0.20, 90% CI [0.04, 0.33]. Specifically, a significant group difference on Appearance Comparison was found, *F*(1, 56) = 9.29, *p* = .004, η²_p_ = 0.14, 90% CI [0.03, 0.29], with the ED group scoring higher (*M* = 5.0, *SD* = 1.5) than the HC group (*M* = 3.7, *SD* = 1.6). No significant group differences were observed for Online Self-Presentation, *F*(1, 56) = 1.97, *p* = .166, η²_p_ = 0.03, 90% CI [0.00, 0.14] (ED: *M* = 5.2, *SD* = 1.2; HC: *M* = 4.8, *SD* = 1.1), or Appearance-Related Activity, *F*(1, 56) = 0.69, *p* = .411, η²_p_ = 0.01, 90% CI [0.00, 0.10] (ED: *M* = 2.4, *SD* = 1.4; HC: *M* = 2.8, *SD* = 1.5; see Fig. 2).

As a subsequent analysis, a MANCOVA with the BSI-18 Global Severity Index as covariate was conducted. The multivariate effect of group, *F*(3, 53) = 3.41, *p* = .024, η²_p_ = 0.16, 90% CI [0.01, 0.29], as well as the group difference on Appearance Comparison remained significant, *F*(1, 55) = 4.24, *p* = .044, η²_p_ = 0.07, 90% CI [0.00, 0.20], while Online Self-Presentation, *F*(1, 55) = 1.44, *p* = .235, η²_p_ = 0.03, 90% CI [0.00, 0.13], and Appearance-Related Activity, *F*(1, 55) = 2.89, *p* = .095, η²_p_ = 0.05, 90% CI [0.00, 0.17], remained non-significant.

#### Correlation analyses

Descriptively, skewness and kurtosis for the SMAPS subscales ranged from − 0.49 to 0.51 and from − 1.06 to 0.04, respectively. Table 3 presents the correlations between the SMAPS subscales and the EDE-Q8, BSI-18, as well as the four engagement items. Bonferroni correction was applied to control for multiple testing (*k* = 27; 3 SMAPS scales × 9 criterion variables).

**Table 3:** Relationship between the SMAPS Scales with EDE-Q8, BSI-18, and Engagement Items (*N* = 58)

Variable	*OS*			*AA*			*AC*		
	*r*_(56)_	*p*	95% CI	*r*_(56)_	*p*	95% CI	*r*_(56)_	*p*	95% CI
EDE-Q8	0.29	0.756	[0.03, 0.51]	0.02	1	[-0.24, 0.28]	0.61	< 0.001	[0.41, 0.75]
BSI-18 (GSI)	0.10	1	[-0.16, 0.35]	0.26	1	[0.01, 0.49]	0.43	0.018	[0.20, 0.62]
Somatization	0.04	1	[-0.22, 0.30]	0.32	0.406	[0.06, 0.53]	0.29	0.693	[0.04, 0.51]
Depression	0.06	1	[-0.20, 0.31]	0.17	1	[-0.10, 0.41]	0.45	0.011	[0.22, 0.63]
Anxiety	0.17	1	[-0.09, 0.41]	0.27	1	[0.01, 0.49]	0.44	0.017	[0.20, 0.62]
Engagement^a^									
Frequency	0.13	1	[-0.20, 0.44]	0.06	1	[-0.27, 0.38]	0.13	1	[-0.21, 0.43]
Editing	0.37	0.665	[0.05, 0.62]	0.17	1	[-0.17, 0.47]	0.23	1	[-0.10, 0.52]
Selection	0.24	1	[-0.09, 0.53]	− 0.07	1	[-0.38, 0.26]	0.14	1	[-0.19, 0.45]
Monitoring	0.40	0.370	[0.09, 0.64]	0.07	1	[-0.26, 0.39]	0.20	1	[-0.13, 0.49]

*p* represents Bonferroni adjusted *p*-values (*p* x 27 tests), capped at 1; OS = Online Self-Presentation, AA = Appearance Related Activity, AC = Appearance Comparison; EDE-Q8 = Eating Disorder Examination – Questionnaire 8; BSI-18 = Brief Symptom Inventory-18; GSI = Global Severity Index. ^a^For engagement with online posting only the 37 participants who reported any posting activity have been analyzed, thus *df* = 35 for these correlations

##### Relationship between the SMAPS and eating disorder symptomatology

Only Appearance Comparison showed a significant positive correlation with eating disorder symptomatology, *r*(56) = 0.61, *p* < .001, 95% CI [0.41, 0.75]. Online Self-Presentation showed a small positive correlation that did not survive Bonferroni correction, *r*(56) = 0.29, *p* = .756, 95% CI [0.03, 0.51], while Appearance-Related Activity was unrelated to eating disorder symptoms, *r*(56) = 0.02, *p* = 1.000, 95% CI [-0.24, 0.28].

##### Relationship between the SMAPS and symptoms of psychopathology

Appearance Comparison showed significant moderate correlations with general psychopathological distress, *r*(56) = 0.43, *p* = .018, 95% CI [0.20, 0.62], with depressive symptoms, *r*(56) = 0.45, *p* = .011, 95% CI [0.22, 0.63], and with anxiety, *r*(56) = 0.44, *p* = .017, 95% CI [0.20, 0.62]. The association with somatization did not reach significance after Bonferroni correction, *r*(56) = 0.29, *p* = .693, 95% CI [0.04, 0.51]. Neither Online Self-Presentation nor Appearance-Related Activity showed significant associations with any BSI-18 scale (all *p* ≥ .406).

##### Relationship between the SMAPS and engagement with online posting

Among active posters, correlations between SMAPS subscales and the four items measuring engagement with online posting did not reach significance (all *p* ≥ .370). Descriptively, Online Self-Presentation showed the highest associations, particularly with monitoring reactions, *r*(35) = 0.40, *p* = .370, 95% CI [0.09, 0.64], and editing, *r*(35) = 0.37, *p* = .665, 95% CI [0.05, 0.62].

## Discussion

The present research aimed to validate a German translation of the SMAPS [38] and to examine whether appearance-related social media behaviors differ between individuals with diagnosed eating disorders and healthy controls across two studies. The three-factor structure of the German SMAPS showed acceptable model fit and significant factor loadings across all items. All subscales demonstrated good internal consistency. The latent correlations among the three factors suggest that the subscales capture related but distinct constructs.

Consistent with prior research [38], Appearance Comparison emerged as the subscale most strongly associated with eating disorder symptomatology (Study 1: *r* = .69; Study 2: *r* = .61). This finding aligns with the central role of appearance-based social comparison in theoretical models linking media exposure to body dissatisfaction and disordered eating [16, 61]. The strength of this association is notable and adds to the growing body of research that has identified comparative evaluation of one’s own appearance against others on social media as particularly relevant mechanism for understanding body image disturbances [6, 62]. Online Self-Presentation and Appearance-Related Activity also showed significant, albeit much smaller, associations with eating disorder symptomatology in Study 1. In Study 2, the association for Online Self-Presentation was of comparable magnitude but did not reach significance, likely due to reduced statistical power, whereas Appearance-Related Activity showed no meaningful association with eating disorder symptomatology in this sample.

Furthermore, Appearance Comparison showed the most robust links to general distress, depressive symptoms, and anxiety, and this pattern was consistent across both studies. This suggests that presenting oneself online and engaging with appearance-related content per se may be less closely tied to psychological distress than the evaluative and comparative processes captured by Appearance Comparison. This finding is consistent with the broader literature linking social comparison processes to negative affective outcomes [38, 42, 63, 64]. In Study 1, Online Self-Presentation was additionally associated with depressive symptoms and Appearance-Related Activity with somatization, but neither association reached significance in Study 2. In addition, all three SMAPS subscales were significantly associated with higher general problematic internet use. Thus, appearance-related social media use may share features with broader patterns of problematic internet use.

In both studies, Online Self-Presentation showed descriptively the strongest associations with the questions that captured personal engagement with online posting, particularly with monitoring others’ reactions to posts, although these associations did not reach significance in Study 2, where the subsample of active posters was considerably smaller. This aligns conceptually with self-presentation theory [65] and the self-presentational motivation underlying this subscale: individuals who are more preoccupied with how they present themselves online are also more likely to invest effort in selecting and editing their posts and to monitor the feedback they receive [11, 12].

The group comparison in Study 2 demonstrated that individuals with eating disorders score significantly higher on Appearance Comparison than healthy controls. While the estimated effect size (η²_p_ = 0.14) corresponds to a large effect, the width of the confidence interval indicates considerable imprecision, and the magnitude of this effect requires confirmation in larger samples. The effect remained significant after controlling for general psychopathology, albeit at a reduced magnitude. The elevated level of appearance comparison in individuals with eating disorders is consistent with the Tripartite Influence Model [16], which identifies appearance-based social comparison as a key mechanism in the development and maintenance of body dissatisfaction. Notably, no significant group differences emerged for Online Self-Presentation or Appearance-Related Activity. However, given the reduced statistical power of Study 2, these null findings may reflect Type II errors rather than evidence for the absence of group differences. Nevertheless, the present findings indicate that it is specifically the comparative dimension of appearance preoccupation on social media that differentiates individuals with eating disorders from healthy controls, but this requires confirmation in adequately powered samples.

The present findings have implications for both future research and clinical practice. The German SMAPS may be a useful tool for assessing appearance-related social media behaviors in research and applied settings. Importantly, Appearance Comparison emerged as the most consistent correlate of eating disorder symptomatology and psychological distress and remained significantly elevated in individuals with eating disorders after controlling for general psychopathology. This suggests that appearance-based social comparison may be a particularly relevant target for prevention and intervention. More broadly, the findings support a shift from global indicators of social media use (e.g., time spent online) toward a process-oriented assessment of how social media is used.

### Limitations

Limitations due to the cross-sectional study designs and study sampling should be considered when interpreting the present findings. First, the use of cross-sectional designs precludes causal inferences about the direction of the observed associations. Longitudinal and experimental designs are needed to establish temporal precedence and examine potential bidirectional relationships between appearance-related social media use and psychological outcomes. Second, both studies included participants that were majority female, young, and well-educated. The small numbers of male and non-binary participants, especially in Study 2, together with the narrow age range, limit conclusions about the applicability of the German SMAPS beyond this demographic and may restrict generalizability to more diverse populations. Relatedly, measurement invariance across gender was not formally tested and should be examined in samples with a larger proportion of men. Third, Study 2 was underpowered relative to the a priori target sample size and the clinical sample was recruited from a specialized eating disorder outpatient clinic, which may limit generalizability to patients treated in other clinical settings. However, there are no strong theoretical reasons to assume that the associations would systematically differ between settings, although this should be examined in future research. Additionally, the inclusion criteria for Study 2 were broadened to also include passive social media users. Although this is a difference to keep in mind, correlations of SMAPS scores with other measures suggest that this had little effect on the results of Study 2 when compared to Study 1. The ED group was also diagnostically heterogeneous. As eating disorder presentations differ in their clinical features and appearance-related concerns, and as the subgroups were too small to permit separate analyses, the findings cannot be attributed to any single ED diagnosis.

Another limitation concerns the fit of the restricted confirmatory model in Study 1, which was acceptable rather than good. Allowing residual covariances, as in the original validation [38], improved model fit. However, as these parameters were identified post hoc, future studies should examine whether a refined item set yields better fit, as some items within the same subscale may overlap in content.

Finally, nearly all participants in Studies 1 and 2 reported predominantly using the same social media platform. No detailed information was collected regarding the specific content or type of use and platforms differ substantially in their visual orientation, interactive affordances, and algorithmic content curation [9, 14, 15]. Thus, future research should examine whether the associations observed in the present study persist when controlling for the use of specific platforms or for distinct features within a given platform.

## Conclusions

In conclusion, the German SMAPS demonstrated acceptable psychometric properties confirming three dimensions of Online Self-Presentation, Appearance-Related Activity, and Appearance Comparison, as originally found in the English version [38]. The German SMAPS subscales also show meaningful associations with heightened symptoms of eating disorders and psychological distress, and more problematic internet use. Across both studies, the Appearance Comparison subscale emerged as the most relevant dimension, showing the strongest and most consistent associations with greater distress and differentiating individuals with eating disorders from healthy controls. Future research should aim to compare individuals across specific forms of eating disorders and in comparison to healthy controls in larger, adequately powered samples; examine the German SMAPS in longitudinal designs to clarify temporal dynamics; and evaluate the utility of the SMAPS in clinical settings for identifying individuals at risk for appearance-related distress in the context of social media use.

## Electronic supplementary material

Below is the link to the electronic supplementary material.

Supplementary Material 1: Supplementary Figure S1: *Three Factor Model of the German Social Media Appearance Preoccupation Scale with four Residual Covariances*.

Supplementary Material 2: German version of the Social Media Appearance Preoccupation Scale (SMAPS).

## Data Availability

The datasets and analysis code supporting the conclusions of this article are available at the Open Science Framework (OSF): https://osf.io/z2scj/ (DOI: 10.17605/OSF.IO/Z2SCJ). To protect participant confidentiality, descriptive sociodemographic variables were removed from the Study 1 dataset, and all sociodemographic variables from the Study 2 dataset given the small clinical sample.

## Abbreviations

SMAPS: Social Media Appearance Preoccupation Scale
OS: Online Self-Presentation
AA: Appearance-Related Activity
AC: Appearance Comparison
EDE-Q8: Eating Disorder Examination-Questionnaire 8
BSI-18: Brief Symptom Inventory-18
GSI: Global Severity Index
GPIUS-2: Generalized Problematic Internet Use Scale 2
POSI: Preference for Online Social Interaction
MR: Mood Regulation
CP: Cognitive Preoccupation
CU: Compulsive Use
NO: Negative Outcome
CFA: Confirmatory Factor Analysis
WLSMV: Weighted Least Squares Mean and Variance Adjusted
RMSEA: Root Mean Square Error of Approximation
SRMR: Standardized Root Mean Square Residual
CFI: Comparative Fit Index
TLI: Tucker-Lewis Index
MANOVA: Multivariate Analysis of Variance
MANCOVA: Multivariate Analysis of Covariance
ED: Eating Disorder
HC: Healthy Control

## References

[1] DataReportal. Digital 2025: Global Overview Report [Internet]. DataReportal – Glob. Digit. Insights. 2025 [cited 2025 Dec 16]. https://datareportal.com/reports/digital-2025-global-overview-report. Accessed 16 Dec 2025.

[2] Cheng X, Fan Y, Li S, Li X, Jin S, Zhou C, et al. Research landscape and trends of internet addiction disorder: A comprehensive bibliometric analysis of publications in the past 20 years. Digit Health. 2025;11:20552076251336940. 10.1177/20552076251336940. 40297375 PMC12034966

[3] Yang Q, Liu J, Rui J. Association between social network sites use and mental illness: A meta-analysis. Cyberpsychology J Psychosoc Res Cyberspace [Internet]. 2022. 10.5817/CP2022-1-1

[4] Ferguson CJ. Do social media experiments prove a link with mental health: A methodological and meta-analytic review. Psychol Pop Media. 2025;14:201–6. 10.1037/ppm0000541.

[5] Saiphoo AN, Vahedi Z. A meta-analytic review of the relationship between social media use and body image disturbance. Comput Hum Behav. 2019;101:259–75. 10.1016/j.chb.2019.07.028.

[6] De Valle MK, Gallego-García M, Williamson P, Wade TD. Social media, body image, and the question of causation: Meta-analyses of experimental and longitudinal evidence. Body Image. 2021;39:276–92. 10.1016/j.bodyim.2021.10.001. 34695681

[7] Holland G, Tiggemann M. A systematic review of the impact of the use of social networking sites on body image and disordered eating outcomes. Body Image. 2016;17:100–10. 10.1016/j.bodyim.2016.02.008. 26995158

[8] Perloff RM. Social Media Effects on Young Women’s Body Image Concerns: Theoretical Perspectives and an Agenda for Research. Sex Roles. 2014;71:363–77. 10.1007/s11199-014-0384-6.

[9] Schmidt J-H, Taddicken M. Soziale Medien: Funktionen, Praktiken, Formationen. In: Schmidt J-H, Taddicken M, editors. Handb Soz Medien. Springer Fachmedien; 2022. pp. 19–34. 10.1007/978-3-658-25995-2_2.

[10] Pedalino F, Camerini A-L. Instagram Use and Body Dissatisfaction: The Mediating Role of Upward Social Comparison with Peers and Influencers among Young Females. Int J Environ Res Public Health. 2022;19:1543. 10.3390/ijerph19031543. 35162562 PMC8834897

[11] Tiggemann M, Anderberg I, Brown Z. Uploading your best self: Selfie editing and body dissatisfaction. Body Image. 2020;33:175–82. 10.1016/j.bodyim.2020.03.002. 32224447

[12] Tiggemann M, Hayden S, Brown Z, Veldhuis J. The effect of Instagram likes on women’s social comparison and body dissatisfaction. Body Image. 2018;26:90–7. 10.1016/j.bodyim.2018.07.002. 30036748

[13] Gill R. Changing the perfect picture: Smartphones, social media and appearance pressures [Internet]. City, University of London; 2021 [cited 2026 Jul 28]. Available from: https://www.citystgeorges.ac.uk/__data/assets/pdf_file/0005/597209/Parliament-Report-web.pdf. Accessed 28 Jul 2026.

[14] Griffiths S, Harris EA, Whitehead G, Angelopoulos F, Stone B, Grey W, et al. Does TikTok contribute to eating disorders? A comparison of the TikTok algorithms belonging to individuals with eating disorders versus healthy controls. Body Image. 2024;51:101807. 10.1016/j.bodyim.2024.101807. 39504757

[15] Harriger JA, Evans JA, Thompson JK, Tylka TL. The dangers of the rabbit hole: Reflections on social media as a portal into a distorted world of edited bodies and eating disorder risk and the role of algorithms. Body Image. 2022;41:292–7. 10.1016/j.bodyim.2022.03.007. 35378338

[16] Thompson JK, Heinberg LJ, Altabe M, Tantleff-Dunn S. Exacting beauty: Theory, assessment, and treatment of body image disturbance. [Internet]. Washington: American Psychological Association; 1999. [cited 2025 Dec 16]. 10.1037/10312-000.

[17] Festinger L, A Theory of Social Comparison Processes. Hum Relat. 1954;7:117–40. . 10.1177/001872675400700202

[18] Tiggemann M, Miller J. The Internet and Adolescent Girls’ Weight Satisfaction and Drive for Thinness. Sex Roles. 2010;63:79–90. 10.1007/s11199-010-9789-z.

[19] Rodgers RF, McLean SA, Paxton SJ. Longitudinal relationships among internalization of the media ideal, peer social comparison, and body dissatisfaction: Implications for the tripartite influence model. Dev Psychol. 2015;51:706–13. 10.1037/dev0000013. 25751099

[20] Van Den Berg P, Thompson JK, Obremski-Brandon K, Coovert M. The Tripartite Influence model of body image and eating disturbance. J Psychosom Res. 2002;53:1007–20. 10.1016/S0022-3999(02)00499-3. 12445590

[21] Fardouly J, Diedrichs PC, Vartanian LR, Halliwell E. Social comparisons on social media: The impact of Facebook on young women’s body image concerns and mood. Body Image. 2015;13:38–45. 10.1016/j.bodyim.2014.12.002. 25615425

[22] Feltman CE, Szymanski DM. Instagram Use and Self-Objectification: The Roles of Internalization, Comparison, Appearance Commentary, and Feminism. Sex Roles. 2018;78:311–24. 10.1007/s11199-017-0796-1.

[23] Hogue JV, Mills JS. The effects of active social media engagement with peers on body image in young women. Body Image. 2019;28:1–5. 10.1016/j.bodyim.2018.11.002. 30439560

[24] Lee M, Lee H-H. Social media photo activity, internalization, appearance comparison, and body satisfaction: The moderating role of photo-editing behavior. Comput Hum Behav. 2021;114:106579. 10.1016/j.chb.2020.106579.

[25] Roberts SR, Maheux AJ, Hunt RA, Ladd BA, Choukas-Bradley S. Incorporating social media and muscular ideal internalization into the tripartite influence model of body image: Towards a modern understanding of adolescent girls’ body dissatisfaction. Body Image. 2022;41:239–47. 10.1016/j.bodyim.2022.03.002. 35306356

[26] Sanzari CM, Gorrell S, Anderson LM, Reilly EE, Niemiec MA, Orloff NC, et al. The impact of social media use on body image and disordered eating behaviors: Content matters more than duration of exposure. Eat Behav. 2023;49:101722. 10.1016/j.eatbeh.2023.101722. 37060807 PMC10363994

[27] Vandenbosch L, Fardouly J, Tiggemann M. Social media and body image: Recent trends and future directions. Curr Opin Psychol. 2022;45:101289. 10.1016/j.copsyc.2021.12.002. 35030460

[28] Engeln R, Loach R, Imundo MN, Zola A. Compared to Facebook, Instagram use causes more appearance comparison and lower body satisfaction in college women. Body Image. 2020;34:38–45. 10.1016/j.bodyim.2020.04.007. 32505866

[29] Cohen R, Newton-John T, Slater A. The relationship between Facebook and Instagram appearance-focused activities and body image concerns in young women. Body Image. 2017;23:183–7. 10.1016/j.bodyim.2017.10.002. 29055773

[30] Jerónimo F, Carraça EV. Effects of fitspiration content on body image: a systematic review. Eat Weight Disord - Stud Anorex Bulim Obes. 2022;27:3017–35. 10.1007/s40519-022-01505-4. PMC9676749 36401082

[31] Lonergan AR, Bussey K, Mond J, Brown O, Griffiths S, Murray SB, et al. Me, my selfie, and I: The relationship between editing and posting selfies and body dissatisfaction in men and women. Body Image. 2019;28:39–43. 10.1016/j.bodyim.2018.12.001. 30572289

[32] Mingoia J, Hutchinson AD, Wilson C, Gleaves DH. The Relationship between Social Networking Site Use and the Internalization of a Thin Ideal in Females: A Meta-Analytic Review. Front Psychol. 2017;8:1351. 10.3389/fpsyg.2017.01351. 28824519 PMC5545754

[33] Fredrickson BL, Roberts T-A. Objectification Theory: Toward Understanding Women’s Lived Experiences and Mental Health Risks. Psychol Women Q. 1997;21:173–206. 10.1111/j.1471-6402.1997.tb00108.x.

[34] Butkowski CP, Dixon TL, Weeks K. Body Surveillance on Instagram: Examining the Role of Selfie Feedback Investment in Young Adult Women’s Body Image Concerns. Sex Roles. 2019;81:385–97. 10.1007/s11199-018-0993-6.

[35] Dakanalis A, Clerici M, Caslini M, Favagrossa L, Prunas A, Volpato C, et al. Internalization of sociocultural standards of beauty and disordered eating behaviours: The role of body surveillance, shame and social anxiety. J Psychopathol. 2014;20:33–7.

[36] Saunders JF, Eaton AA. Snaps, Selfies, and Shares: How Three Popular Social Media Platforms Contribute to the Sociocultural Model of Disordered Eating Among Young Women. Cyberpsychology Behav Soc Netw. 2018;21:343–54. 10.1089/cyber.2017.0713. 29883209

[37] Orben A. Teenagers, screens and social media: a narrative review of reviews and key studies. Soc Psychiatry Psychiatr Epidemiol. 2020;55:407–14. 10.1007/s00127-019-01825-4. 31925481

[38] Zimmer-Gembeck MJ, Hawes T, Pariz J. A closer look at appearance and social media: Measuring activity, self-presentation, and social comparison and their associations with emotional adjustment. Psychol Pop Media. 2021;10:74–86. 10.1037/ppm0000277.

[39] Firasta L, Vani MF, Lucibello KM, Sabiston CM. Understanding social media appearance preoccupation: The role of body image emotions. Psychol Pop Media. 2025;14:472–7. 10.1037/ppm0000559.

[40] Zimmer-Gembeck MJ, Hawes T, Scott RA, Campbell T, Webb HJ. Adolescents’ online appearance preoccupation: A 5-year longitudinal study of the influence of peers, parents, beliefs, and disordered eating. Comput Hum Behav. 2023;140:107569. 10.1016/j.chb.2022.107569.

[41] Zimmer-Gembeck MJ, Scott RA, Hawes T. Appearance-related teasing, rejection sensitivity, acceptance, and coping as risks and resources associated with online appearance preoccupation over one year. Comput Hum Behav. 2024;158:108319. 10.1016/j.chb.2024.108319.

[42] Zimmer-Gembeck MJ, Seekis V, Duffy AL. Online appearance preoccupation in and beyond adolescence: A longitudinal study of social media use, anxiety, and depression as correlates of growth and stability. Psychol Pop Media. 2026;15:11–21. 10.1037/ppm0000587.

[43] Leiner DJ. SoSci Survey [Internet]. 2024. https://www.soscisurvey.de

[44] Comrey AL, Lee HB. A first course in factor analysis. 2nd ed. Lawrence Erlbaum Associates; 1992.

[45] Muthén LK, Muthén BO. How to Use a Monte Carlo Study to Decide on Sample Size and Determine Power. Struct Equ Model Multidiscip J. 2002;9:599–620. 10.1207/S15328007SEM0904_8.

[46] Schmitt M, Eid M. Richtlinien für die Übersetzung fremdsprachlicher Messinstrumente. Diagnostica. 2007;53:1–2. 10.1026/0012-1924.53.1.1.

[47] Kliem S, Mößle T, Zenger M, Strauß B, Brähler E, Hilbert A. The eating disorder examination-questionnaire 8: A brief measure of eating disorder psychopathology (EDE‐Q8). Int J Eat Disord. 2016;49:613–6. 10.1002/eat.22487. 26711183

[48] Hilbert A, Tuschen-Caffier B, Karwautz A, Niederhofer H, Munsch S. Eating Disorder Examination-Questionnaire. Diagnostica. 2007;53:144–54. 10.1026/0012-1924.53.3.144.

[49] Spitzer C, Hammer S, Löwe B, Grabe H, Barnow S, Rose M, et al. Die Kurzform des Brief Symptom Inventory (BSI – 18): erste Befunde zu den psychometrischen Kennwerten der deutschen Version. Fortschr Neurol · Psychiatr. 2011;79:517–23. 10.1055/s-0031-1281602. 21870312

[50] Franke GH, Jaeger S, Glaesmer H, Barkmann C, Petrowski K, Braehler E. Psychometric analysis of the brief symptom inventory 18 (BSI-18) in a representative German sample. BMC Med Res Methodol. 2017;17:14. 10.1186/s12874-016-0283-3. 28125960 PMC5270206

[51] Caplan SE. Theory and measurement of generalized problematic Internet use: A two-step approach. Comput Hum Behav. 2010;26:1089–97. 10.1016/j.chb.2010.03.012.

[52] Barke A, Nyenhuis N, Kröner-Herwig B. The German Version of the Generalized Pathological Internet Use Scale 2: A Validation Study. Cyberpsychology Behav Soc Netw. 2014;17:474–82. 10.1089/cyber.2013.0706. 24742070

[53] R Core Team. R: A language and environment for statistical computing. Available from: https://www.R-project.org/

[54] Brauer K, Ranger J, Ziegler M. Confirmatory Factor Analyses in Psychological Test Adaptation and Development: A Nontechnical Discussion of the WLSMV Estimator. Psychol Test Adapt Dev. 2023;4:4–12. 10.1027/2698-1866/a000034.

[55] Hu L, Bentler PM. Cutoff criteria for fit indexes in covariance structure analysis: Conventional criteria versus new alternatives. Struct Equ Model Multidiscip J. 1999;6:1–55. 10.1080/10705519909540118.

[56] Schermelleh-Engel K, Moosbrugger H, Müller H. Evaluating the Fit of Structural Equation Models: Tests of Significance and Descriptive Goodness-of-Fit Measures [Internet]. 2003. 10.23668/PSYCHARCHIVES.12784

[57] Bentler PM, Bonett DG. Significance tests and goodness of fit in the analysis of covariance structures. Psychol Bull. 1980;88(3):588–606. 10.1037/0033-2909.88.3.588.

[58] George D, Mallery P. IBM SPSS Statistics 23 step by step: a simple guide and reference. 14th ed. New York: Routledge; 2016.

[59] Armstrong RA. When to use the Bonferroni correction. Ophthalmic Physiol Opt. 2014;34:502–8. 10.1111/opo.12131. 24697967

[60] Champely S, Ekstrom C, Dalgaard P, Gill J, Weibelzahl S, Anandkumar A et al. Package ‘pwr’: Basic Functions for Power Analysis. 2020. https://github.com/heliosdrm/pwr

[61] So B, Kwon KH. The Impact of Thin-Ideal Internalization, Appearance Comparison, Social Media Use on Body Image and Eating Disorders: A Literature Review. J Evid-Based Soc Work. 2023;20:55–71. 10.1080/26408066.2022.2117582.

[62] Seeto C-J, Uhlmann LR, Zimmer-Gembeck MJ, Donovan CL. Vulnerability to appearance-based social media use and preoccupation: A model of young women’s appearance values, depression, and self-esteem via uses and gratification. Body Image. 2026;56:102034. 10.1016/j.bodyim.2026.102034. 41621366

[63] Aubry R, Quiamzade A, Meier LL. Depressive symptoms and upward social comparisons during Instagram use: A vicious circle. Personal Individ Differ. 2024;217:112458. 10.1016/j.paid.2023.112458.

[64] McComb CA, Vanman EJ, Tobin SJ. A Meta-Analysis of the Effects of Social Media Exposure to Upward Comparison Targets on Self-Evaluations and Emotions. Media Psychol. 2023;26:612–35. 10.1080/15213269.2023.2180647.

[65] Baumeister RF. A self-presentational view of social phenomena. Psychol Bull. 1982;91:3–26. 10.1037/0033-2909.91.1.3.

