# Supplementary material for: Appearance-related social media behavior and eating disorder pathology: evidence from the german social media appearance preoccupation scale in nonclinical and clinical samples
Source: J Eat Disord. 2026 Sep 5;14:211. doi: 10.1186/s40337-026-01762-z (PMC13548597; doi:10.1186/s40337-026-01762-z)
Supplement: Supplementary file 1 [file 40337_2026_1762_MOESM1_ESM.docx]

**Supplementary Material 1: Three Factor Model of the German Social Media Appearance Preoccupation Scale with four Residual Covariances**

**Supplementary Figure S1**

*Three Factor Model of the German Social Media Appearance Preoccupation Scale with four Residual Covariances*

*Note*. OS = Online Self-Presentation; AA = Appearance-Related Activity; AC = Appearance Comparison; Circles represent latent variables, squares refer to manifest variables, single-headed arrows represent standardized factor loadings, double-headed arrows between latent variables represent standardized latent correlations, double-headed arrows between manifest variables represent standardized residual covariances; error terms of manifest variables are not shown; all factor loadings, latent correlations, and residual covariances are significant at *p* < .001.
