# Supplementary material for: Appearance-related social media behavior and eating disorder pathology: evidence from the german social media appearance preoccupation scale in nonclinical and clinical samples
Source: J Eat Disord. 2026 Sep 5;14:211. doi: 10.1186/s40337-026-01762-z (PMC13548597; doi:10.1186/s40337-026-01762-z)
Supplement: Supplementary file 2 [file 40337_2026_1762_MOESM2_ESM.docx]

## Supplementary Material 2: German version of the Social Media Appearance Preoccupation Scale (SMAPS)

*Instruction:* Bitte kreuzen Sie an, wie sehr Sie den Aussagen zustimmen.

*Online Self-Presentation*:

1. Ich ziehe es vor, nur Fotos von mir in soziale Medien hochzuladen, auf denen ich körperlich attraktiv aussehe.
2. Ich ziehe es vor, nur Fotos von mir in soziale Medien hochzuladen, auf denen ich fit und gesund aussehe.
3. Wenn andere Fotos von mir in soziale Medien hochladen, konzentriere ich mich darauf, ob ich gut ausgesehen habe.
4. Ich überprüfe, wer die Fotos von mir oder meinem Körper in sozialen Medien kommentiert, liked oder anschaut.
5. Wenn andere Fotos von mir in soziale Medien hochladen, rege ich mich auf, wenn ich nicht besonders gut aussehe.
6. Ich stimme Fotos von mir selbst zu, bevor sie jemand mit einem Tag versehen kann.
7. Wenn ich Fotos von mir selbst hochlade, verwende ich normalerweise Filter oder bearbeite bzw. verändere sie, um mich besser aussehen zu lassen.

*Appearance-Related Activity:*

8. Wenn meine Freund:innen in sozialen Medien sind, posten, kommentieren, teilen oder liken sie Inhalte darüber, wie man fit und/oder muskulös wird oder bleibt.
9. Wenn meine Freund:innen in sozialen Medien sind, posten, kommentieren, teilen oder liken sie Inhalte darüber, was und wann man essen sollte.
10. Wenn ich in sozialen Medien bin, poste, kommentiere, teile oder like ich Inhalte darüber, wie man fit und/oder muskulös wird oder bleibt.
11. Wenn ich in sozialen Medien bin, poste, kommentiere, teile oder like ich Inhalte darüber, was und wann man essen sollte.
12. Wenn meine Freund:innen in sozialen Medien sind, posten, kommentieren, teilen oder liken sie Inhalte darüber, wie sie ihren Körper gerne hätten.

*Appearance Comparison:*

13. Ich bin oft unzufrieden mit meinem Gewicht oder meinem Aussehen auf meinen Bildern in sozialen Medien.
14. Im Vergleich zu meinen Freund:innen in sozialen Medien fühle ich mich in meiner äußerlichen Erscheinung unzulänglich.
15. Ich habe das Gefühl, dass ich meine Ernährung ändern möchte, nachdem ich die Bilder anderer Leute online gesehen habe.
16. Wie ich mich in Bezug auf meinen Körper und mein Aussehen fühle, wird von den Bildern anderer Menschen in den sozialen Medien beeinflusst.
17. Wenn ich Bilder von anderen sehe, fühle ich mich eher niedergeschlagen.
18. Ich habe das Gefühl, dass ich meine Trainingsroutine oder mein Fitnesslevel ändern möchte, nachdem ich die Bilder anderer Leute online gesehen habe.

*Scale: 1 = stimme überhaupt nicht zu bis 7 = stimme voll und ganz zu*

*For the original English version please refer to*:

Zimmer-Gembeck MJ, Hawes T, Pariz J. A closer look at appearance and social media: Measuring activity, self-presentation, and social comparison and their associations with emotional adjustment. Psychol Pop Media. 2021;10:74–86. https://doi.org/10.1037/ppm0000277
